# Centromere-specific histone Cse4 by the chaperone Scm3

**DOI:** 10.1186/1756-8935-6-S1-P95

**Published:** 2013-03-18

**Authors:** Uhn-Soo Cho, Stephen C Harrison

**Affiliations:** 1 Department of Biological Chemistry, University of Michigan, Ann Arbor, Ml 48109, USA; 2 Department of Biological Chemistry and Molecular Pharmacology and Howard Hughes Medical Institute, Harvard Medical School, Boston, MA 02115, USA

## 

A specialized nucleosome is a component of all eukaryotic kineto-chores. The core of this nucleosome contains a centromere-specific histone, CENP-A (the Cse4 gene product in budding yeast), instead of the usual H3. Assembly of a centromeric nucleosome depends on a specific chaperone, called Scm3 in yeast and HJURP in higher eukaryotes. We describe here the structure of a complex formed by an N-terminal fragment of Scm3 with the histone-fold domains of Cse4, and H4, all prepared as recombinant proteins derived from the budding yeast *Kluyveromyces lactis*. The contacts of Scm3 with Cse4 explain its selectivity for the centromere-specific histone; key residues at the interface are conserved in HJURP, indicating a common mechanism for centromeric histone deposition. We also report the structure of a (Cse4:H4)_2_ heterotetramer; comparison with the structure of the Scm3:Cse4:H4 complex shows that tetramer formation and DNA binding require displacement of Scm3 from the nucleosome core. The two structures together suggest that specific contacts between the chaperone and Cse4, rather than an altered overall structure of the nucleosome core, determine the selective presence of Cse4 at centromeres.

